# Interleukin-18 mediates cardiac dysfunction induced by western diet independent of obesity and hyperglycemia in the mouse

**DOI:** 10.1038/nutd.2017.1

**Published:** 2017-04-10

**Authors:** S Carbone, P J H Lee, A G Mauro, E Mezzaroma, R Buzzetti, B Van Tassell, A Abbate, S Toldo

**Affiliations:** 1 VCU Pauley Heart Center, Virginia Commonwealth University, Richmond, VA, USA; 2 Victoria Johnson Research Laboratories, Virginia Commonwealth University, Richmond, VA, USA; 3 Department of Experimental Medicine, Sapienza University of Rome, Rome, Italy; 4 School of Pharmacy, Virginia Commonwealth University, Richmond, VA, USA

## Abstract

Obesity and diabetes are independent risk factors for heart failure and are associated with the consumption of diet rich in saturated fat and sugar, Western diet (WD), known to induce cardiac dysfunction in the mouse through incompletely characterized inflammatory mechanisms. We hypothesized that the detrimental cardiac effects of WD are mediated by interleukin-18 (IL-18), pro-inflammatory cytokine linked to cardiac dysfunction. C57BL/6J wild-type male mice and IL-18 knockout male mice were fed high-saturated fat and high-sugar diet for 8 weeks. We measured food intake, body weight and fasting glycemia. We assessed left ventricular (LV) systolic and diastolic function by Doppler echocardiography and cardiac catheterization. In wild-type mice, WD induced a significant increase in isovolumetric relaxation time, myocardial performance index and left ventricular end-diastolic pressure, reflecting an impairment in diastolic function, paired with a mild reduction in LV ejection fraction. IL-18 KO mice had higher food intake and greater increase in body weight without significant differences in hyperglycemia. Despite displaying greater obesity, IL-18 knockout mice fed with WD for 8 weeks had preserved cardiac diastolic function and higher left ventricular ejection fraction. IL-18 mediates diet-induced cardiac dysfunction, independent of food intake and obesity, thus highlighting a disconnect between the metabolic and cardiac effects of IL-18.

## Introduction

The epidemic in obesity and diabetes has been linked to the consumption of a diet rich in saturated fat and sugars, Western diet (WD), which is by itself able to induce obesity, diabetes, and cardiac systolic and diastolic dysfunction in animals.^1^ The molecular mechanisms involved in the diet-induced cardiac dysfunction are largely unknown.

Systemic inflammation negatively affects cardiac function, and predicts exercise capacity and hospital readmission in patients with heart failure.^2, 3^ The pro-inflammatory interleukin (IL)-18 is a cytokine of the interleukin-1 family that is abundant in the heart and is involved in the development and progression of cardiac hypertrophy, remodeling and failure.^4, 5^ *In vitro* studies suggest that specific nutrients such as saturated fat and sugars can increase the production of IL-18 through direct activation of the intracellular macromolecular complex Nod-like receptor pyrin domain-containing protein 3 (NLRP3) inflammasome.^6, 7, 8, 9, 10, 11, 12^ IL-18, on the other hand, also regulates hunger and satiety, body weight and composition, and glucose metabolism, and it opposes obesity and insulin resistance.^13^ IL-18 may therefore represent the missing link between a pro-inflammatory diet, that is, WD, and cardiac dysfunction.^1, 14^ In this study, we measured cardiac and systemic effects of WD in mice with IL-18 genetic deletion (IL-18 KO) testing two competing hypotheses, one by which deletion of IL-18 would promote greater food intake and weight gain, thus enhancing the negative impact of WD on cardiac function, and the alternative hypothesis by which deletion of IL-18 will protect the heart from the negative effects of WD, thus preserving cardiac function.

## Materials and methods

Experiments were conducted in 8- to 10-week old male genetically modified mice lacking the gene for IL-18 (IL-18 knockout (KO) mice) (Jackson Laboratories, Bar Harbor, ME, USA) and age-matched C57BL/6J wild-type (WT) male mice (Envigo, Madison, WI, USA). A group of age-matched male CD1 WT mice (Envigo) was used to measure IL-18 mRNA in the heart (real-time PCR, SYBR Green PCR master mix; Life Technologies, Carlsbad, CA, USA) and IL-18 serum levels (ELISA, Quant; Bio-Tek, Winooski, VT, USA) after 8 weeks of WD (TD.88137; Envigo) compared with Standard diet (Teklad LM-485; Envigo).

We fed IL-18 KO and WT mice with WD (TD.88137; Envigo) providing unlimited access to food.^1^ Food intake was measured daily, body weight weekly. Capillary glucose was obtained from the tail at baseline and 8 weeks (Clarity Diagnostics, Boca Raton, FL, USA), after 8–10 h fasting.

Mice underwent transthoracic Doppler echocardiography (Vevo770; VisualSonics, Toronto, ON, Canada) under sedation (30–50 mg kg^−1^ pentobarbital) at baseline, 4 and 8 weeks.^1^ Left ventricular (LV) catheterization with a retrograde right carotid artery approach (Millar Inc., Houston, TX, USA) to measure LV end-diastolic pressure (LVEDP) was performed at 8 weeks under anesthesia (50–70 mg kg^−1^ pentobarbital), prior to killing.^1^ The hearts were explanted to measure LV interstitial fibrosis using Masson's trichrome (Sigma-Aldrich, St Louis, MO, USA) computer morphometric analysis (Image ProPlus 6.0 software, Media Cybernetics, Rockville, MD, USA).^1^ Values were expressed as the mean and standard error of the mean. The statistical analysis was performed using SPSS 22.0 (IBM Corp., Armonk, NY, USA). Differences within each group or between groups were analyzed using the Student's *T*-test for paired and unpaired data, respectively. A sample size of 6 or more for each group provided a power >80% (*α*=0.05) to detect a meaningful difference defined as difference between means exceeding the standard deviation in each group. No animals or samples were excluded from the data analysis. The investigators performing echocardiography or samples analyses were blinded to the treatment allocations and genotype of the animals. The investigator feeding and handling mice kept other investigators blinded to the treatment and the genotype. No randomization was performed. All studies were approved by the Virginia Commonwealth University Institutional Animal Care and Use Committee and conducted according to the NIH guidelines.

## Results

**Figure 1:**
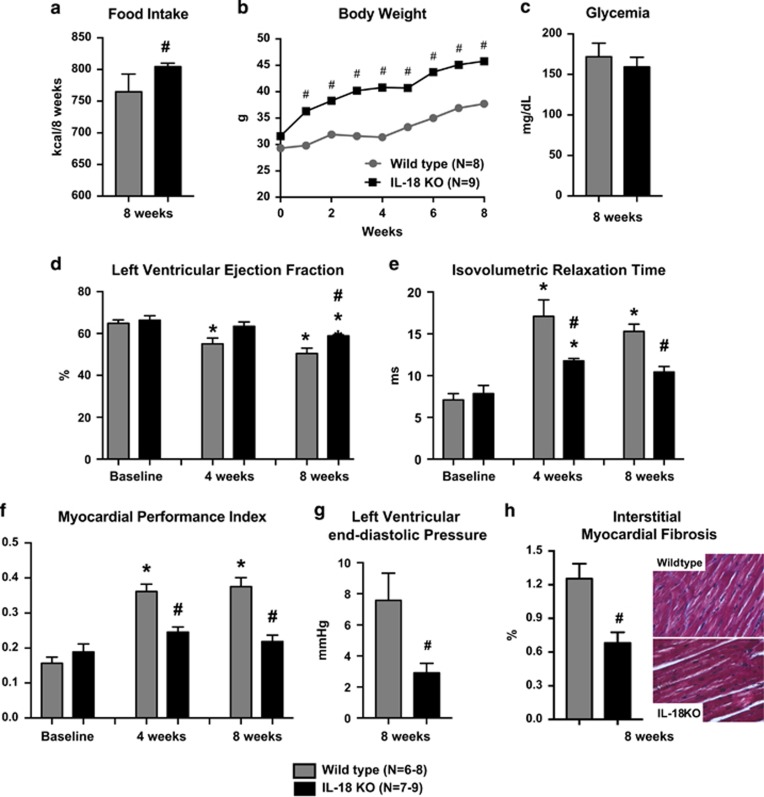
Interleukin-18 (IL-18) paradox in diet-induced obesity and cardiac dysfunction. Interleukin-18 (IL-18) knockout (KO) mice had a higher food intake (**a**) and body weight (**b**), and similar glucose (**c**) compared with wild type. IL-18 KO mice presented partially preserved systolic (**d**) and diastolic function (**e**–**g**) and less myocardial interstitial fibrosis (**h**). ^#^*P*<0.05 vs wild type; *P<0.05 vs Baseline.

Compared with mice fed standard diet, WD significantly increased IL-18 plasma levels (+35%, *P*=0.01) and IL-18 cardiac mRNA expression (+127%, *P*=0.04) (Supplementary Figure S1). There were no significant differences in cardiac or metabolic parameters between IL-18 KO and WT mice at baseline (Supplementary Figure S2). IL-18 KO mice on WD had a higher 8-week cumulative caloric intake (804±2 vs 765±10 kcal, *P*<0.001; Figure 1a) and weight gain (+38.8% vs +20.0%, *P*<0.001; Figure 1b), without significant differences in fasting glycemia (152±32 vs 184±49 mg dl^−1^, *P*=0.13; Figure 1c).

Despite higher caloric intake and weight gain, IL-18 KO mice showed a significantly greater preservation of LV ejection fraction (Figure 1d), reflecting better systolic function, and shorter isovolumetric relaxation time (Figure 1e), lower myocardial performance index (Figure 1f) and lower LVEDP (Figure 1g), reflecting better diastolic function compared with WT mice fed with the same WD. Myocardial interstitial fibrosis was also significantly less in KO compared with WT mice (Figure 1h).

## Discussion

These results show the complexity of IL-18 signaling in cardiac function and metabolism. WD impairs cardiac function and promotes obesity through distinct signaling pathways. IL-18 mediates cardiac systolic and diastolic dysfunction induced by WD, providing the possible missing link between the two. IL-18, however, does not mediate the impairment in metabolism nor promotes weight gain seen with WD, while it actually appears to limit these changes.^13^ The disconnect between body weight and cardiac function in IL-18 KO mice fed with WD suggests the existence of an 'IL-18-paradox*'* possibly due to the presence of two different signaling pathways: one leading to cardiac dysfunction and dependent upon IL-18 and one leading to weight gain which is independent of, and actually improved by, IL-18.^13^ Mice with genetic deletion of Nod-like receptor pyrin domain-containing protein 3 (NLRP3), the sensor in the NLRP3 inflammasome responsible for the processing of IL-18 and of IL-1β, and mice with genetic deletion of IL-1 have preserved cardiac function and reduced weight gain compared with wild-type mice fed WD,^15, 16, 17, 18^ thus suggesting a hierarchy in the response to systemic inflammation by which IL-1β mediates the cardiac effects through activation of IL-18 in the heart^5^ and a systemic response that is, at least in part, independent of IL-18 in this model.

**Figure 2:**
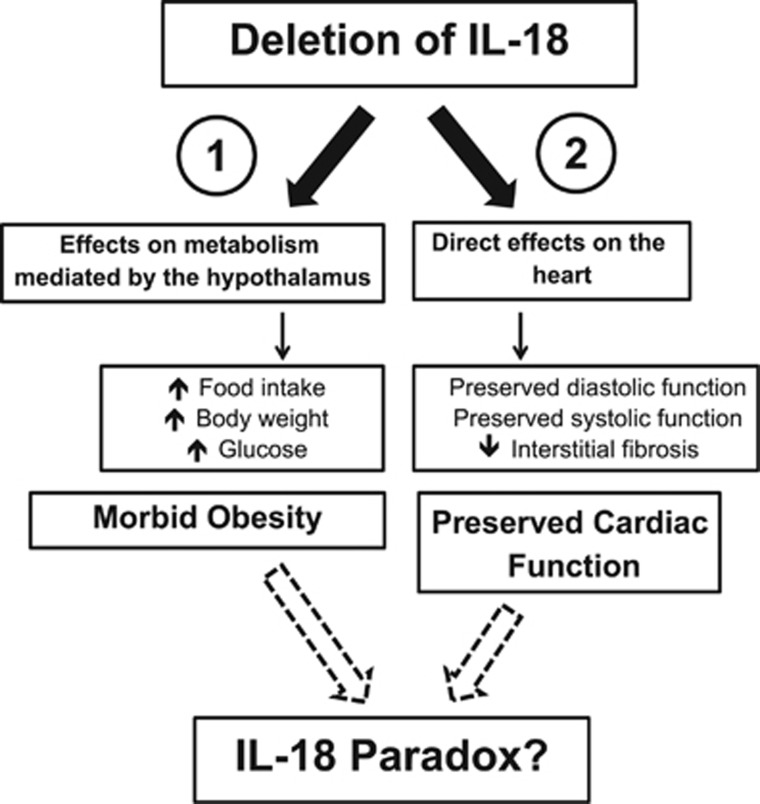
Disconnect between the effects of IL-18 deletion on the heart and on metabolism. The disconnect between the effects of IL-18 deletion on the heart and on metabolism suggests the presence of two possible independent pathways: (1) a metabolic pathway, mediated by the central nervous system (CNS), with negative effects of IL-18 deletion on metabolism by increasing food intake, body weight and glucose and (2) a cardiac pathway, with direct protective effect of IL-18 deletion on the heart function by improvement of systolic and diastolic function, and reducing interstitial fibrosis.

This may occur through two different signaling pathways, one in the brain regulating food intake^13^ and one in the heart regulating cardiac function^4^ (Figure 2). Systemically administered IL-18, indeed, impairs cardiac function,^5^ but does not affect food intake,^13^ and IL-18 limits food intake only if administered directly in the brain.^13^

The association of obesity and metabolic abnormalities with HF is object of intense investigation, particularly suggesting that obesity causes HF ('obesity cardiomyopathy') and that weight loss would cure HF.^19^ However, obesity may also be considered a co-morbid condition that further limits exercise capacity in HF without directly affecting cardiac function.^20^

IL-18 KO mice had better cardiac function despite greater weight gain, showing that in response to WD, obesity and cardiac dysfunction occur simultaneously, yet the former is not the cause for the latter.

The observation that IL-18 KO mice are obese yet have better cardiac function than the leaner WT mice is vaguely reminiscent of the 'obesity paradox' in patients with HF, by which patients with HF and are obese tend to have a more favorable prognosis.^21, 22^

From a translational standpoint, IL-18-targeted strategies may be valuable in preventing or treating heart failure.^4, 23^ An IL-18-blocking antibody and recombinant IL-18 binding protein, which binds circulating IL-18 preventing it from binding to the membrane receptor, are being developed as clinical therapeutics. Of note, in preclinical studies^5, 22^ and in phase I and II clinical trials,^24, 25^ these inhibitors did not impair metabolism nor induce weight gain. This is in contrast with the data from the IL-18 KO mouse model.^13^ This substantial difference likely lies in the different IL-18 signaling in the brain, with the IL-18 KO mouse lacking IL-18 throughout the body, including the brain, whereas the antibody and the binding protein fail to cross the blood brain barrier and penetrate the brain and hence have no effect on food intake and weight gain. Similar findings would be expected to be seen in obese subjects, in which we believe that treatment with IL-18-binding protein or IL-18 antibody would still exert beneficial effects on the heart, without inducing an increase in food intake, body weight and impairment in glucose metabolism. A recent pilot study in obese subjects with type 2 diabetes treated with IL-18 antibody for 12 weeks has shown a safe profile of the drug, tested at different doses, without impairing glucose metabolism.^24^

In our study, mice were fed WD for 8 weeks based on our prior study where 4 and 8 weeks of WD induced a significant cardiac dysfunction.^1^ We cannot exclude that the cardiac dysfunction induced by WD and the protective effects of IL-18 deletion would differ if mice were fed high-saturated fat and high-sugar diet for a longer period, in which metabolic abnormalities will likely become more prominent. Moreover, we have not measured the levels of other cytokines that may also be involved in the cardiac dysfunction induced by WD and potentially be affected by genetic deletion of IL-18. However, in a prior study the preservation of cardiac function seen in IL-18 KO mouse or after IL-18 blockade therapy did not affect IL-6 plasma levels, suggesting that the protective effects on the heart were independent of IL-6.^5^

Finally, we did not measure the level of oxidative stress induced by high-sugar and high-saturated fat diet, which has also been reported to affect the heart by directly impairing cardiac contractile function,^26^ and how the genetic deletion of IL-18 affected such parameters.

In conclusion, IL-18 mediates the cardiac dysfunction induced by a high-saturated fat and high-sugar diet, independent of food intake, obesity and hyperglycemia, showing a disconnect between diet-induced obesity, altered metabolism and cardiac dysfunction.

## References

[bib1] Carbone S, Mauro AG, Mezzaroma E, Kraskauskas D, Marchetti C, Buzzetti R et al. A high-sugar and high-fat diet impairs cardiac systolic and diastolic function in mice. Int J Cardiol 2015; 198: 66–69. 26151718 10.1016/j.ijcard.2015.06.136

[bib2] Van Tassell BW, Toldo S, Mezzaroma E, Abbate A. Targeting interleukin-1 in heart disease. Circulation 2013; 128: 1910–1923. 24146121 10.1161/CIRCULATIONAHA.113.003199 PMC3938092

[bib3] Heymans S, Hirsch E, Anker SD, Aukrust P, Balligand J-L, Cohen-Tervaert JW et al. Inflammation as a therapeutic target in heart failure? A scientific statement from the Translational Research Committee of the Heart Failure Association of the European Society of Cardiology. Eur J Heart Fail 2009; 11: 119–129. 19168509 10.1093/eurjhf/hfn043 PMC2639409

[bib4] O'Brien LC, Mezzaroma E, Van Tassell BW, Marchetti C, Carbone S, Abbate A et al. Interleukin-18 as a therapeutic target in acute myocardial infarction and heart failure. Mol Med 2014; 20: 221–229. 24804827 10.2119/molmed.2014.00034 PMC4069269

[bib5] Toldo S, Mezzaroma E, O'Brien L, Marchetti C, Seropian IM, Voelkel NF et al. Interleukin-18 mediates interleukin-1-induced cardiac dysfunction. Am J Physiol Hear Circ Physiol 2014; 306: 1025–1031. 10.1152/ajpheart.00795.2013 PMC3962640 24531812

[bib6] Wen H, Gris D, Lei Y, Jha S, Zhang L, Huang MT-H et al. Fatty acid-induced NLRP3-ASC inflammasome activation interferes with insulin signaling. Nat Immunol 2011; 12: 408–415. 21478880 10.1038/ni.2022 PMC4090391

[bib7] Reynolds CM, McGillicuddy FC, Harford KA, Finucane OM, Mills KHG, Roche HM. Dietary saturated fatty acids prime the NLRP3 inflammasome via TLR4 in dendritic cells-implications for diet-induced insulin resistance. Mol Nutr Food Res 2012; 56: 1212–1222. 22700321 10.1002/mnfr.201200058

[bib8] Li X, Du N, Zhang Q, Li J, Chen X, Liu X et al. MicroRNA-30d regulates cardiomyocyte pyroptosis by directly targeting foxo3a in diabetic cardiomyopathy. Cell Death Dis 2014; 5: e1479. 25341033 10.1038/cddis.2014.430 PMC4237254

[bib9] Collino M, Benetti E, Rogazzo M, Mastrocola R, Yaqoob MM, Aragno M et al. Reversal of the deleterious effects of chronic dietary HFCS-55 intake by PPAR-δ agonism correlates with impaired NLRP3 inflammasome activation. Biochem Pharmacol 2013; 85: 257–264. 23103566 10.1016/j.bcp.2012.10.014

[bib10] Stienstra R, Joosten LAB, Koenen T, van Tits B, van Diepen JA, van den Berg SAA et al. The inflammasome-mediated caspase-1 activation controls adipocyte differentiation and insulin sensitivity. Cell Metab 2010; 12: 593–605. 21109192 10.1016/j.cmet.2010.11.011 PMC3683568

[bib11] L'homme L, Esser N, Riva L, Scheen A, Paquot N, Piette J et al. Unsaturated fatty acids prevent activation of NLRP3 inflammasome in human monocytes/macrophages. J Lipid Res 2013; 54: 2998–3008. 24006511 10.1194/jlr.M037861 PMC3793604

[bib12] Sui Y-H, Luo W-J, Xu Q-Y, Hua J. Dietary saturated fatty acid and polyunsaturated fatty acid oppositely affect hepatic NOD-like receptor protein 3 inflammasome through regulating nuclear factor-kappa B activation. World J Gastroenterol 2016; 22: 2533–2544. 26937141 10.3748/wjg.v22.i8.2533 PMC4768199

[bib13] Netea MG, Joosten LA, Lewis E, Jensen DR, Voshol PJ, Kullberg BJ et al. Deficiency of interleukin-18 in mice leads to hyperphagia, obesity and insulin resistance. Nat Med 2006; 12: 650–656. 16732281 10.1038/nm1415

[bib14] Carbone S, Shah KB, Van Tassell B, JMJ Canada, Evans RK, Regan JA et al. Obesity and diastolic heart failure: is inflammation the link? Transl Med 2013; 3: e124.

[bib15] Vandanmagsar B, Youm Y-H, Ravussin A, Galgani JE, Stadler K, Mynatt RL et al. The NLRP3 inflammasome instigates obesity-induced inflammation and insulin resistance. Nat Med 2011; 17: 179–188. 21217695 10.1038/nm.2279 PMC3076025

[bib16] Mcgillicuddy FC, Harford KA, Reynolds CM, Oliver E, Claessens M, Mills KHG et al. Lack of interleukin-1 receptor I (IL-1RI) protects mice from high-fat diet-induced adipose tissue inflammation coincident with improved glucose homeostasis. Diabetes 2011; 60: 1688–1698. 21515850 10.2337/db10-1278 PMC3114387

[bib17] Matsuki T, Horai R, Sudo K, Iwakura Y. IL-1 plays an important role in lipid metabolism by regulating insulin levels under physiological conditions. J Exp Med 2003; 198: 877–888. 12975454 10.1084/jem.20030299 PMC2194201

[bib18] Coll RC, LAJO Neill, Schroder K. Questions and controversies in innate immune research: what is the physiological role of NLRP3? Nat Publ Gr 2016; 1–5. 10.1038/cddiscovery.2016.19 PMC4979470 27551512

[bib19] Kitzman DW, Shah SJ. The HFpEF obesity phenotype: The elephant in the room. J Am Coll Cardiol 2016; 68: 200–203. 27386774 10.1016/j.jacc.2016.05.019

[bib20] Carbone S, Canada JM, Buckley L, Trankle CR, Dixon DL, Buzzetti R et al. Obesity contributes to exercise intolerance in heart failure with preserved ejection fraction. J Am Coll Cardiol 2016; 68: 2487–2488. 27908355 10.1016/j.jacc.2016.08.072 PMC5748881

[bib21] Lavie CJ, Alpert MA, Arena R, Mehra MR, Milani RV, Ventura HO. Impact of obesity and the obesity paradox on prevalence and prognosis in heart failure. JACC Heart Fail 2013; 1: 93–102. 24621833 10.1016/j.jchf.2013.01.006

[bib22] Carbone S, Lavie CJ, Arena R et al. Obesity and Heart Failure: Focus on the Obesity Paradox. Mayo Clinic Proceedings 2017. In press. Available at http://dx.doi.org/10.1016/j.mayocp.2016.11.001. 10.1016/j.mayocp.2016.11.001 28109619

[bib23] Regan JA, Mauro AG, Carbone S, Marchetti C, Mezzaroma E, Krauskaus D et al. Interleukin-18 blockade prevents diastolic dysfunction in a mouse model of heart failure with preserved ejection fraction. Circulation 2015; 132: A14648–A14648.

[bib24] McKie EA, Reid JL, Mistry PC, DeWall SL, Abberley L, Ambery PD et al. A study to investigate the efficacy and safety of an anti-interleukin-18 monoclonal antibody in the treatment of type 2 diabetes mellitus. PLoS One 2016; 11: e0150018. 26930607 10.1371/journal.pone.0150018 PMC4773233

[bib25] Tak PP, Bacchi M, Bertolino M. Pharmacokinetics of IL-18 binding protein in healthy volunteers and subjects with rheumatoid arthritis or plaque psoriasis. Eur J Drug Metab Pharmacokinet 31: 109–116. 16898079 10.1007/BF03191127

[bib26] Tsutsui H, Kinugawa S, Matsushima S. Oxidative stress and heart failure. AJP Heart Circ Physiol 2011; 301: H2181–H2190. 10.1152/ajpheart.00554.2011 21949114

