# Supplementary material for: Interleukin-18 mediates cardiac dysfunction induced by western diet independent of obesity and hyperglycemia in the mouse
Source: Nutr Diabetes. 2017 Apr 10;7(4):e258–. doi: 10.1038/nutd.2017.1 (PMC5436096; doi:10.1038/nutd.2017.1)
Supplement: Supplementary Figure 1 [file nutd20171x1.ppt]

## Slide 1
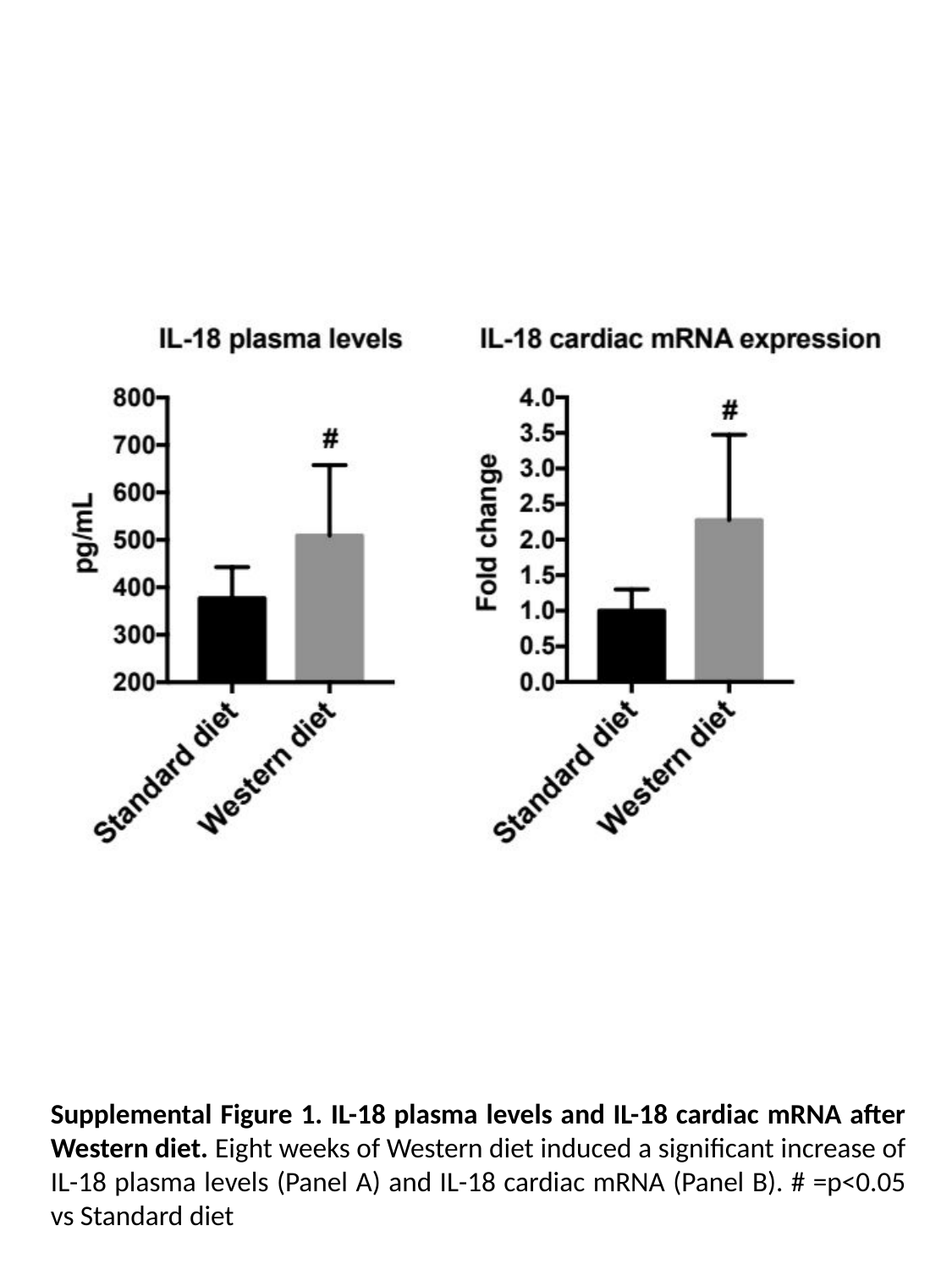

Supplemental Figure 1. IL-18 plasma levels and IL-18 cardiac mRNA after Western diet. Eight weeks of Western diet induced a significant increase of IL-18 plasma levels (Panel A) and IL-18 cardiac mRNA (Panel B). # =p<0.05 vs Standard diet
