# Supplementary material for: Interleukin-18 mediates cardiac dysfunction induced by western diet independent of obesity and hyperglycemia in the mouse
Source: Nutr Diabetes. 2017 Apr 10;7(4):e258–. doi: 10.1038/nutd.2017.1 (PMC5436096; doi:10.1038/nutd.2017.1)
Supplement: Supplementary Figure 2 [file nutd20171x2.ppt]

## Slide 1
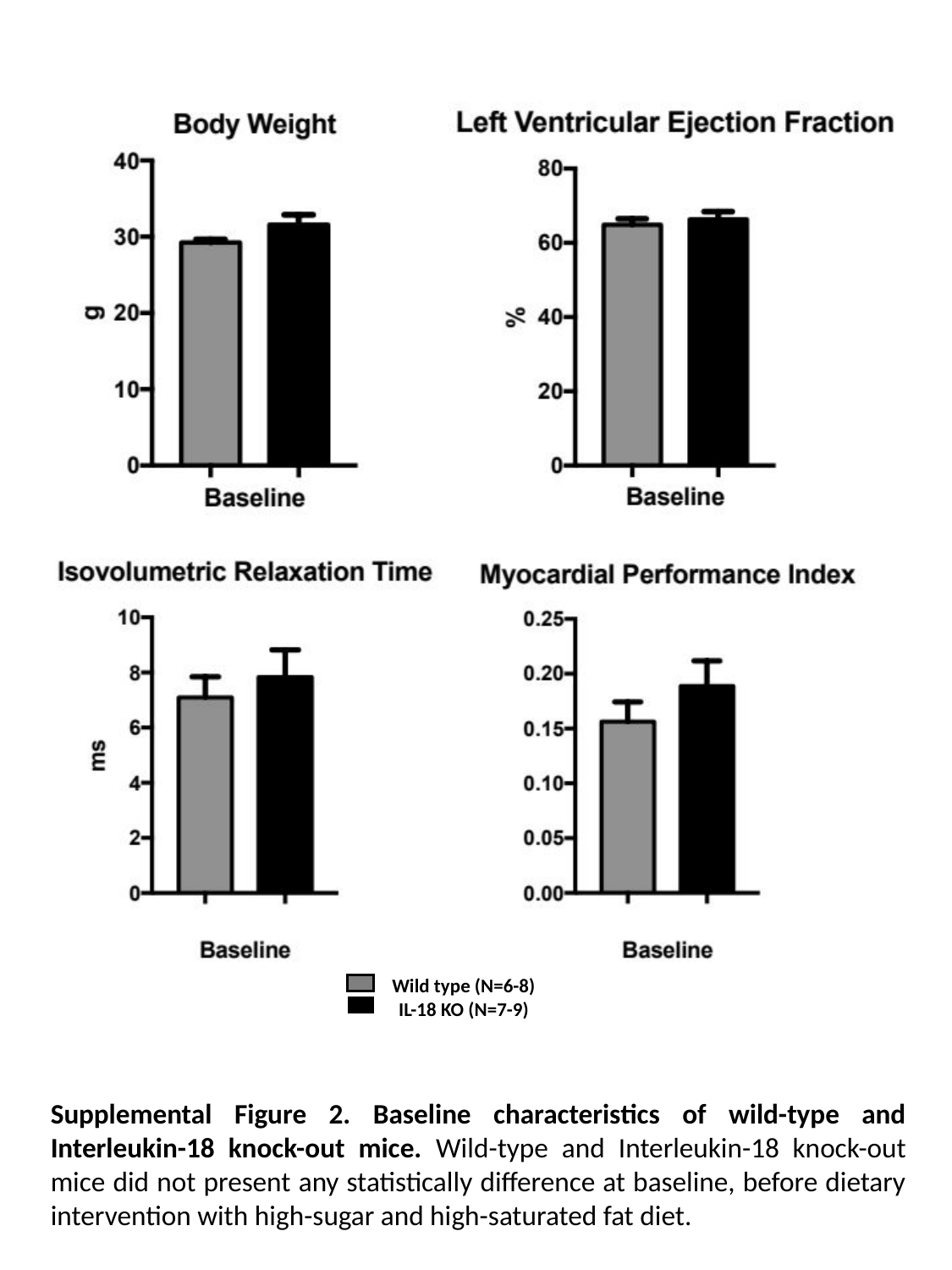

Wild type (N=6-8)
IL-18 KO (N=7-9)
Supplemental Figure 2. Baseline characteristics of wild-type and Interleukin-18 knock-out mice. Wild-type and Interleukin-18 knock-out mice did not present any statistically difference at baseline, before dietary intervention with high-sugar and high-saturated fat diet.
